# Salinomycin is effective for clinically relevant radioresistant oral squamous cell carcinoma cancer cells and their cancer stem cell marker positive fraction

**DOI:** 10.3892/mi.2026.335

**Published:** 2026-08-11

**Authors:** Ryo Saga, Yoichiro Hosokawa, Manabu Fukumoto

**Affiliations:** 1 Department of Radiation Science, Hirosaki University Graduate School of Health Sciences, Hirosaki, Aomori 036-8564, Japan; 2 Department of Rehabilitation Science, Hirosaki University of Health and Welfare, Hirosaki, Aomori 036-8102, Japan; 3 Radiological Disasters and Medical Science Laboratory, Disaster Medical Science Division, International Research Institute of Disaster Science, Tohoku University, Sendai, Miyagi 980-8572, Japan

**Keywords:** radioresistance, cancer stem cell, salinomycin, cell sorter, oral squamous cell carcinoma

## Abstract

Numerous patients with cancer experience metastasis, recurrence and resistance to chemotherapy and radiotherapy due to the presence of cancer stem cells (CSCs). Salinomycin (Sal) is a candidate for CSC-targeting therapy and may consequently sensitize tumors to radiation. Therefore, the present study investigated whether Sal can alleviate radioresistance using a clinically relevant radioresistant cell line and sorted CSC-like cells. The human oral squamous carcinoma cell line, SAS, and its radioresistant counterpart, SAS-R, were used. The fraction of CD24^-^/CD44^+^ cells was identified in the CSC-like cell population and that of CD24^+^/CD44^+^ cells was in identified in the non-CSC-like counterparts. These were sorted from the SAS cells. Clonogenic potency was evaluated using a colony formation assay. The positive fractions of CSC markers (CD24 and CD44) and the number of apoptotic SAS and SAS-R cells were measured using flow cytometry. The percentage of the CD24^+^/CD44^+^ fraction in the SAS-R cells was lower than that in the SAS cells, whereas the CD24^-^/CD44^+^ population was higher in the SAS-R cells than in the SAS cells. The Sal concentration-dependent decrease in the cell surviving fraction was more rapid in the SAS-R cells than in the SAS cells (IC50 values: 0.250 and 0.332 µM, respectively). The fraction of CD24^-^/CD44^+^ cells exhibited a greater radioresistance than the parent SAS cells and the fraction of CD24^+^/CD44^+^ cells. Moreover, the surviving fraction of CD24^-^/CD44^+^ cells was significantly reduced by treatment with 0.3 µM Sal. The addition of Sal increased the number of apoptotic cells compared to 2 Gy radiation alone. On the whole, the present study indicates that Sal is effective in killing the radioresistant population in SAS cells, which comprise a large proportion of CSCs in oral cancer, that is, the CD24-/CD44^+^ fraction. Furthermore, clinically relevant radioresistant cells (SAS-R) are sensitive to Sal; therefore, further investigations are necessary.

## Introduction

The treatment of cancer includes surgery, radiation, chemotherapy and immunotherapy, and the efficacy of these treatments continues to improve. However, cancer is the second leading cause of mortality, and numerous patients with cancer succumb to the disease due to metastasis, recurrence, and resistance to drugs and radiation (1,2). Cancer characteristics, such as metastasis, recurrence and acquired resistance are considered to be caused by cancer stem cells (CSCs) within tumors (3). Shibue and Weinberg (4) indicated that conventional treatments are not sufficiently effective against CSCs and that recurrence due to CSCs is a serious issue.

Currently, patients with cancer receive external X-ray beam radiotherapy during the course of their treatment (5). Despite the implementation of novel treatment strategies, resistance to radiotherapy and disease recurrence remain the major limitations in radiation oncology. CSC radioresistance is a major cause of the failure of radiotherapy and tumor recurrence, even following radiotherapy (6). Oral cancer is one of the most prevalent types of cancer and causes of cancer-related mortality worldwide. Oral squamous cell carcinoma (OSCC) is the most frequently diagnosed form of oral cancer (7).

Despite advances in radiotherapy, the prognosis of patients with OSCC has not improved over the past 30 years, and the acquisition of radioresistance during fractionated irradiation is considered to be one of the reasons for the poor prognosis (8). In a previous study, the prognostic analysis of patients with advanced-stage OSCC treated with intra-arterial chemoradiotherapy revealed that the 5-year survival, disease-free survival and local control rates were 64.5, 59.9 and 85.5%, respectively (9). These results suggested that metastasis by CSCs reduced the survival rate, although a high control rate was achieved (9).

Salinomycin (Sal) has been widely used as an antibiotic in livestock and has been shown to exert anticancer effects. Its effectiveness against CSCs and anticancer drug-resistant cells has particularly attracted attention (10). Gupta *et al* (11) were the first to demonstrate its efficacy against CSCs. They screened chemicals against breast CSCs and concluded that Sal was the most effective (11). It has been demonstrated that Sal suppresses CSCs in a number of types of cancer, including breast cancer, neuroblastoma, glioblastoma, medulloblastoma, pancreatic cancer, colon cancer, prostate cancer, melanoma and lung cancer (12).

However, studies on radiotherapy combined with Sal have not yet been conducted for OSCC, at least to the best of our knowledge. Radioresistant cell lines, such as oral squamous carcinoma SAS-R cells, can be experimentally established following fractionated X-ray irradiation at 2 Gy/day for >30 days, which is often performed as a standard radiotherapy plan (13). The present study examined the effects of Sal to sort these radioresistant cells.

## Materials and methods

### Reagents and antibodies

Sal from *Streptomyces albus* was purchased from MilliporeSigma (cat. no. S4526), and dissolved in dimethyl sulfoxide (DMSO, Wako Pure Chemical Industries, Ltd.). Phycoerythrin (PE)-conjugated monoclonal mouse anti-human CD44 (cat. no. 338803) and mouse IgG1, κ isotype control (cat. no. 400114), as well as fluorescein isothiocyanate (FITC)-conjugated monoclonal mouse anti-human CD24 (cat. no. 311104) and mouse IgG2a, isotype control (cat. no. 400208) were purchased from BioLegend, Inc.

### Cells and cell culture

The human oral squamous carcinoma cell line, SAS, and its radioresistant counterpart, SAS-R, were used. These cell lines were obtained from the Cell Resource Center for Biomedical Research, Institute of Development, Aging and Cancer, Tohoku University. It should be noted that the SAS-R cells acquired radioresistance following exposure to 2 Gy of X-rays daily for >30 days *in vitro*, as previously described (13). These cell lines were maintained at 37˚C in a 5% CO_2_ environment in Roswell Park Memorial Institute (RPMI)-1640 medium (Thermo Fisher Scientific, Inc.) supplemented with 10% heat-inactivated fetal bovine serum (FBS; Japan Bio Serum) and 1% penicillin/streptomycin (Life Technologies; Thermo Fisher Scientific, Inc.).

### Irradiation conditions

In a single-dose experiment, the cultured cells were irradiated with kilovoltage X-rays (150 kVp, 1.0 Gy/min) through an additional 0.5 mm aluminum and 0.3 mm copper filter using an X-ray generator (MBR-1520R-3; Hitachi Medical Co., Ltd.). The concertation in the air was monitored using a thimble ionization chamber placed next to the sample during irradiation. The uncertainty of the absorbed dose measured by the thimble ionization chamber was ±1%.

### Colony formation assay

The clonogenic potency was evaluated using a colony formation assay. Seeded cells were allowed to attach to the bottom of the culture dish for 2 h, and then Sal was administered at concentrations of 0, 0.01, 0.03, 0.1, 0.3, 0.6 and 1 µM 1 h prior to irradiation. For the experiment involving combined treatment with 2 Gy, the 0.01 µM concentration was excluded as it was not expected to affect the surviving fraction. The cells were fixed with methanol at room temperature for 1 min (Wako Pure Chemical Industries, Ltd.) 7-10 days following irradiation and stained with Giemsa staining solution at room temperature for 1 h (Wako Pure Chemical Industries, Ltd.). Colonies containing >50 cells were counted under a microscope (IX71N-22PH, Olympus Corporation). The surviving fraction for each cell line was calculated as the ratio of the plating efficiency of irradiated cells to that of non-irradiated cells.

### Flow cytometric analysis for detecting CSC marker-positive fractions

The CSC marker-positive fractions of SAS and SAS-R cells were measured by flow cytometry using a FACS Aria flow cytometer (BD Biosciences). The present study used CD24 and CD44, which are CSC markers for head and neck cancer. Trypsinized cells were adjusted to a density of 1x10^6^ cells/ml and washed twice with phosphate-buffered saline without calcium and magnesium chloride [PBS(-), Takara Bio, Inc.]. The cells were then incubated with FITC-CD24 (3 µl/10^6^ cells) and PE-CD44 (3 µl/10^6^ cells) or respective isotype control antibodies (3 µl/10^6^ cells) for 15 min at 4˚C in the dark. The cells were then washed twice with PBS(-) containing 5% FBS and analyzed. The CD24^+^/CD44^+^ and CD24^-^/CD44^+^ fractions were sorted based on the isotype control measurement.

### Spheroid assays

Cells (1.0x10^3^ cells/dish) were plated on a φ35 low-adhesive dish (PrimeSurface^®^; Sumitomo Bakelite Co., Ltd.) in serum-free RPMI-1640 supplemented with epidermal growth factor (10 ng/ml; Wako Pure Chemical Industries, Ltd.) and basic fibroblast growth factor (10 ng/ml; Wako Pure Chemical Industries, Ltd.). The cells were treated with Sal (0.3 µM). Spheroid formation was observed over a 1-week period. Spheroid formation was assessed in the microscopic field of view using an Olympus microscope (Olympus Corporation) equipped with a TH4-100 light source and 4X objective. Spheroids >50 µm in length (n=3 dishes) were counted for each of the treatment groups.

### Apoptotic cell detection

Apoptotic cells were detected using FITC-conjugated Annexin V (cat. no. 640906, BioLegend, Inc.) and 7-AAD (cat. no. 420404, BioLegend, Inc.). Trypsinized cells were adjusted to a density of 1x10^6^ cells/ml and washed with PBS(-); the cells were then incubated for 20 min at 4˚C in the dark following the addition of FITC-Annexin V (5 µl/10^6^ cells) and 7-AAD (10 µl/10^6^ cells) and analyzed using a FACS Aria flow cytometer (BD Biosciences). The percentage of FITC-Annexin V-positive cells was defined as the percentage of apoptotic cells, i.e., FITC-Annexin V(+)/7-AAD(-) fraction and FITC-Annexin V(+)/7-AAD(+) fraction.

### Statistical analysis

The experiment was repeated three times. The value of 50% inhibition concentration (IC50) was calculated by fitting the results of colony formation assay using log-logistic regression model. The significance of the differences between the non-treatment and treatment (i.e., irradiation and/or Sal) groups was evaluated using the Tukey-Kramer post-hoc test following two-way analysis of variance. These analyses were performed using Microsoft Excel 2010 (Microsoft Corporation). A value of P<0.05 was considered to indicate a statistically significant difference.

## Results

**Figure 1:**
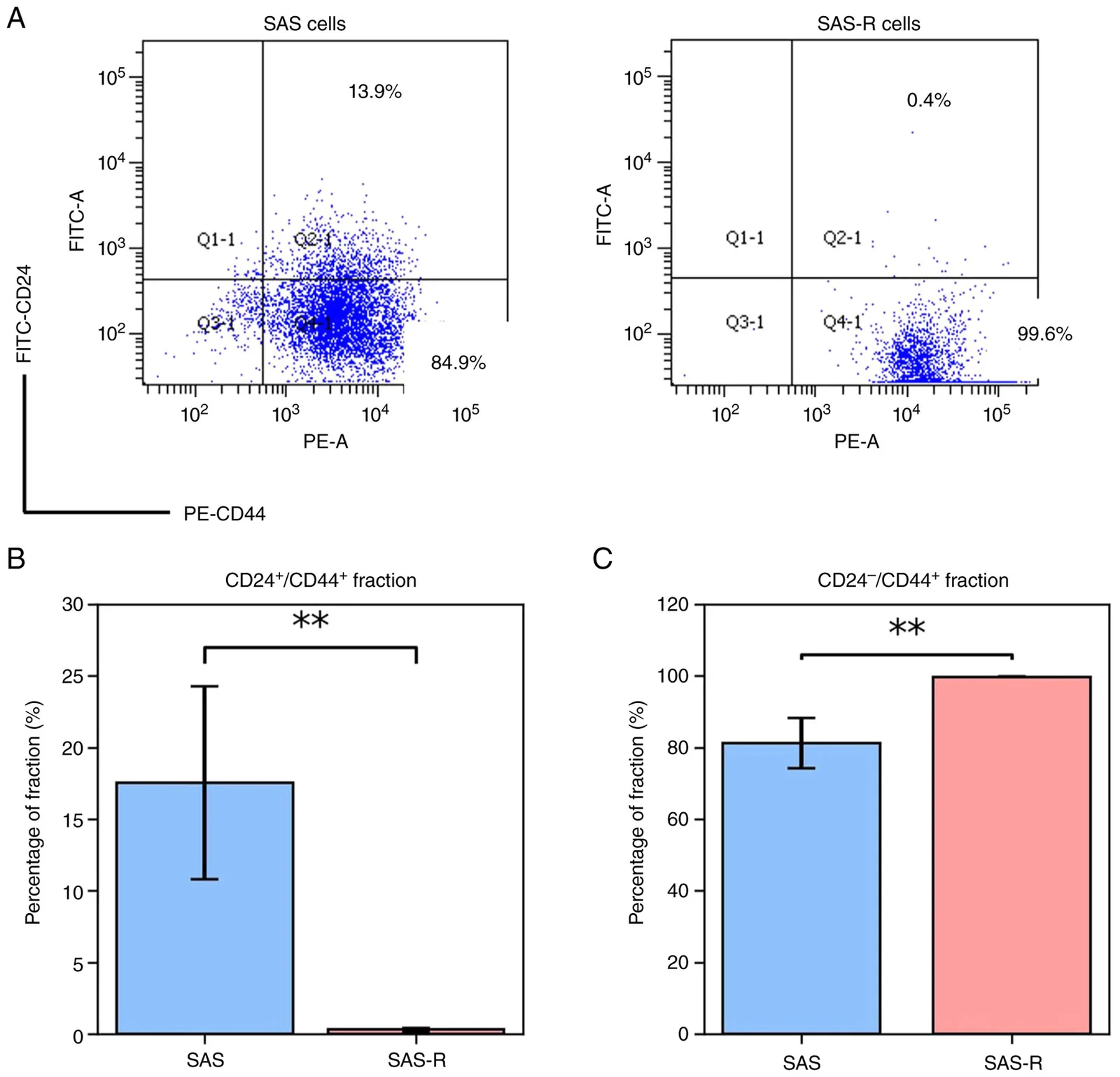
Measurements of the cancer stem cell markers, CD24 and CD44. (A) Representative cytograms of SAS and SAS-R cells. The fractions of (B) CD24^+^/CD44^+^ and (C) CD24^-^/CD44^+^ were measured from the fractions shown in the cytograms. Values are presented as the mean ± standard deviation. ^**^P<0.01, significant differences.

The CSC-like fractions of the SAS and SAS-R cells analyzed for CD24 and CD44 are presented in Fig. 1. The percentage of the CD24^+^/CD44^+^ fraction in the SAS-R cells was significantly lower than that in the SAS cells (0.30±0.10% vs. 17.53±6.73%, P<0.01). The percentage of the CD24^-^/CD44^+^ fraction was higher in the SAS-R cells compared with that in the SAS cells (99.70±0.1 vs. 81.17±6.99, P<0.01), indicating an expanded population of the CSC-like fraction in the SAS-R cells compared with that in the SAS cells.

**Figure 2:**
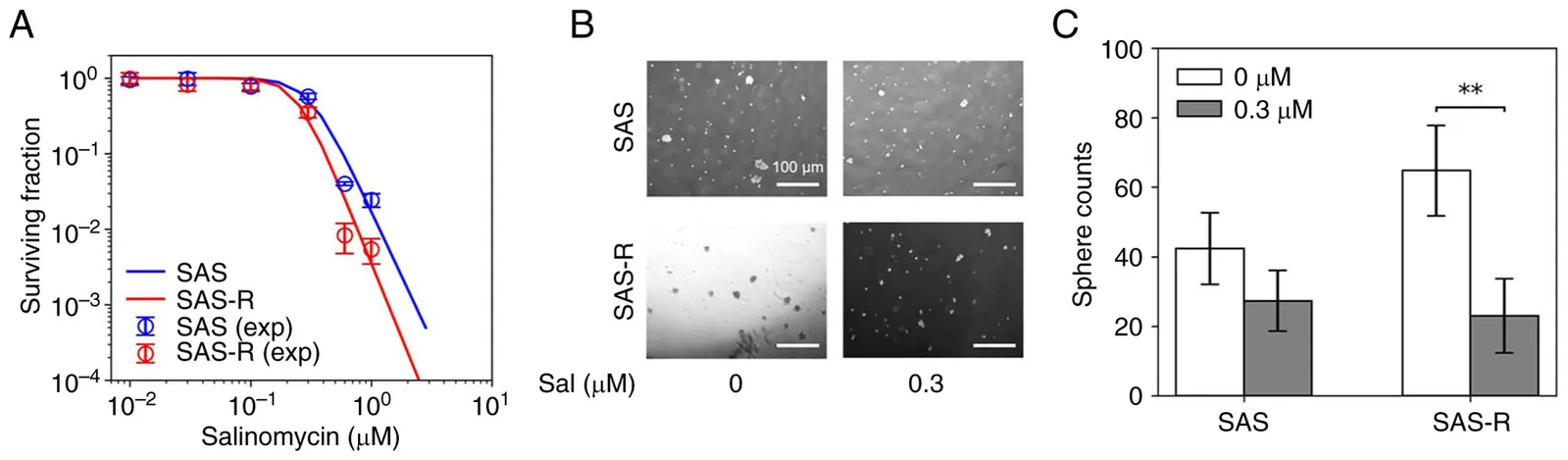
Cell surviving fraction and sphere formation following treatment with Sal. (A) The cell surviving fraction of the SAS and SAS-R cells, Sal concentration (i.e., 0, 0.01, 0.03, 0.1, 0.3, 0.6 and 1 µM) gradient. Exp represents experimental data. Blue and Red solid lines indicate log-logistic sigmoidal curve fitting SAS and SAS-R cell surviving fraction, respectively. (B) Representative images are shown. White scale bars, 100 µm. (C) Sphere counts following treatment with 0.3 µM Sal. Values are presented as the mean ± standard deviation. ^**^P<0.01, significant differences. Sal, salinomycin.

The results of colony formation and spheroid assays following treatment with Sal at the concentration of 0, 0.01, 0.03, 0.1, 0.3, 0.6 and 1 µM are illustrated in Fig. 2. The surviving fraction of SAS and SAS-R cells decreased in a concentration-dependent manner following treatment with Sal (Fig. 2A). The IC50 value calculated from the log-logistic model of the SAS and SAS-R cells was 0.332 and 0.250 µM, respectively. The addition of 0.3 µM Sal, which was a concentration near IC50 for both cell lines, resulted in a significant decrease in the sphere formation of SAS-R cells compared with that of the controls (Fig. 2B and C, P<0.01).

**Figure 3:**
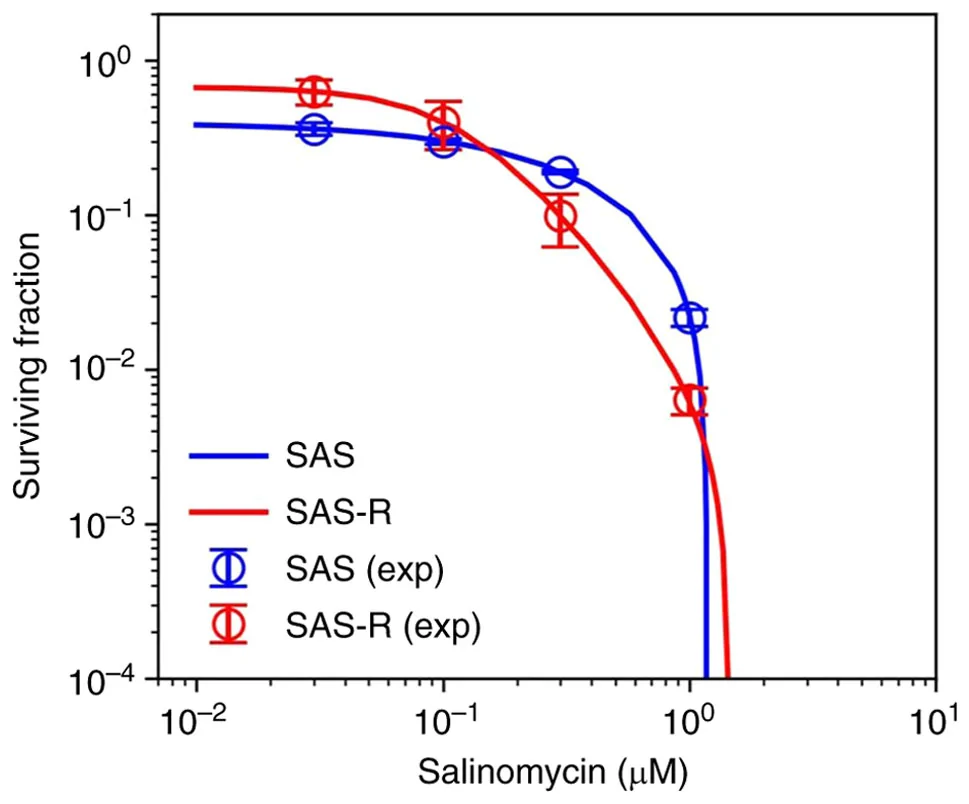
Surviving fractions of SAS and SAS-R cells following 2 Gy irradiation under Sal administration. These surviving fractions were normalized to the respective untreated controls for SAS (blue) and SAS-R (red) cells. Exp represents experimental data.

The cell surviving fraction following 2 Gy irradiation was evaluated under the Sal concentrations 0, 0.03, 0.1, 0.3, 0.6, and 1 µM (Fig. 3). The surviving fraction was significantly lower in the SAS-R cells than in the SAS cells (Fig. 3). These results indicate that Sal can efficiently kill SAS-R cells in which the CSC-like population is expanded.

**Figure 4:**
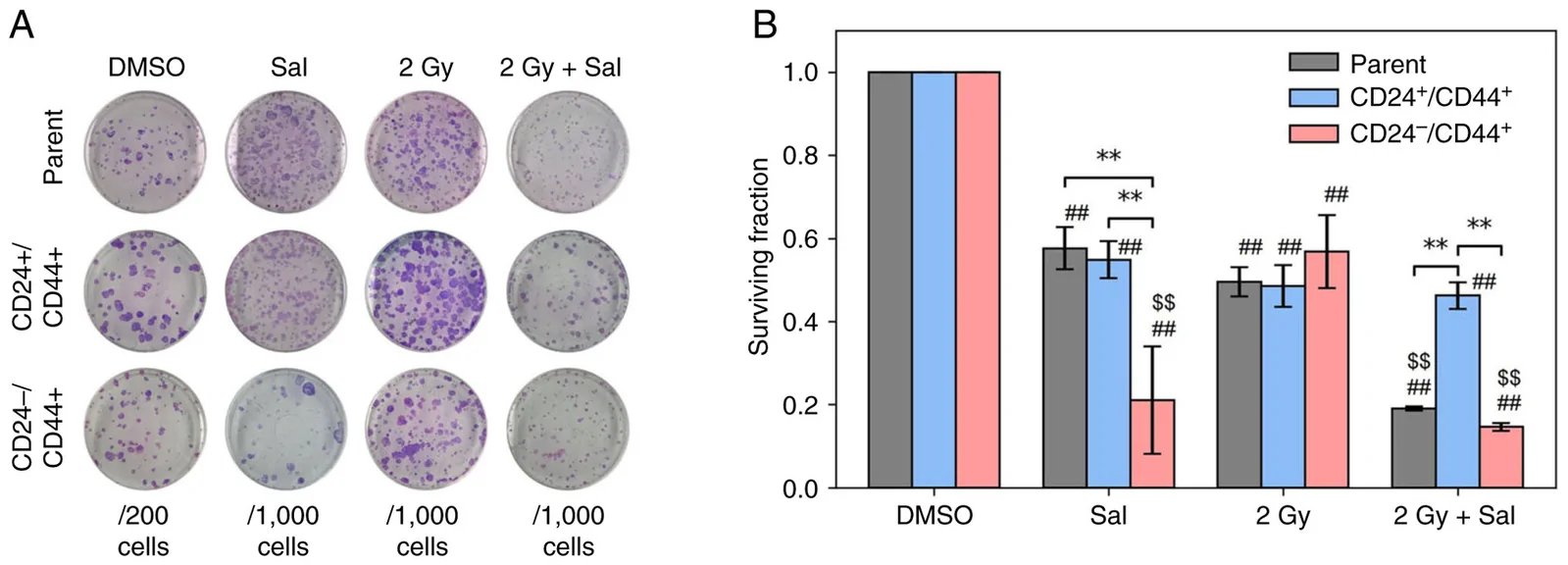
Cell surviving fractions of SAS cells and their subpopulations. (A) Representative colony images are shown. The numbers indicate the number of seeded cells. (B) Surviving fractions of the parental SAS cells and their subpopulations (i.e., CD24^+^/CD44^+^ and CD24^-^/CD44^+^ fractions). Values are presented as the mean ± standard deviation. ^**^P<0.01, ^##^P<0.01 and ^$$^P<0.01, significant differences between brackets (above the bars), DMSO group and 2 Gy group, respectively. Sal, salinomycin.

To demonstrate that Sal was effective against CSC-like fractions, the CD24^-^/CD44^+^ and CD24^+^/CD44^+^ fractions were sorted from the parental SAS cells, and the sensitivity of Sal was measured using a colony formation assay (Fig. 4). Two-way ANOVA revealed a significant interaction between Sal treatment and irradiation in all three cell populations (parent, P<0.05; CD24^-^/CD44^+^, P<0.01; and CD24^+^/CD44^+^, P<0.01). The CD24^-^/CD44^+^ cells tended to exhibit a higher surviving fraction following irradiation alone than the parental and CD24^+^/CD44^+^ cells. By contrast, treatment with Sal significantly decreased the surviving fraction of the CD24^-^/CD44^+^ cells compared with the other fractions. Taken together, these results suggest that the CSC-like CD24^-^/CD44^+^ fraction is highly sensitive to Sal treatment and exhibits a more pronounced reduction in surviving fraction following the combined treatment with irradiation and Sal than the other cell fractions.

**Figure 5:**
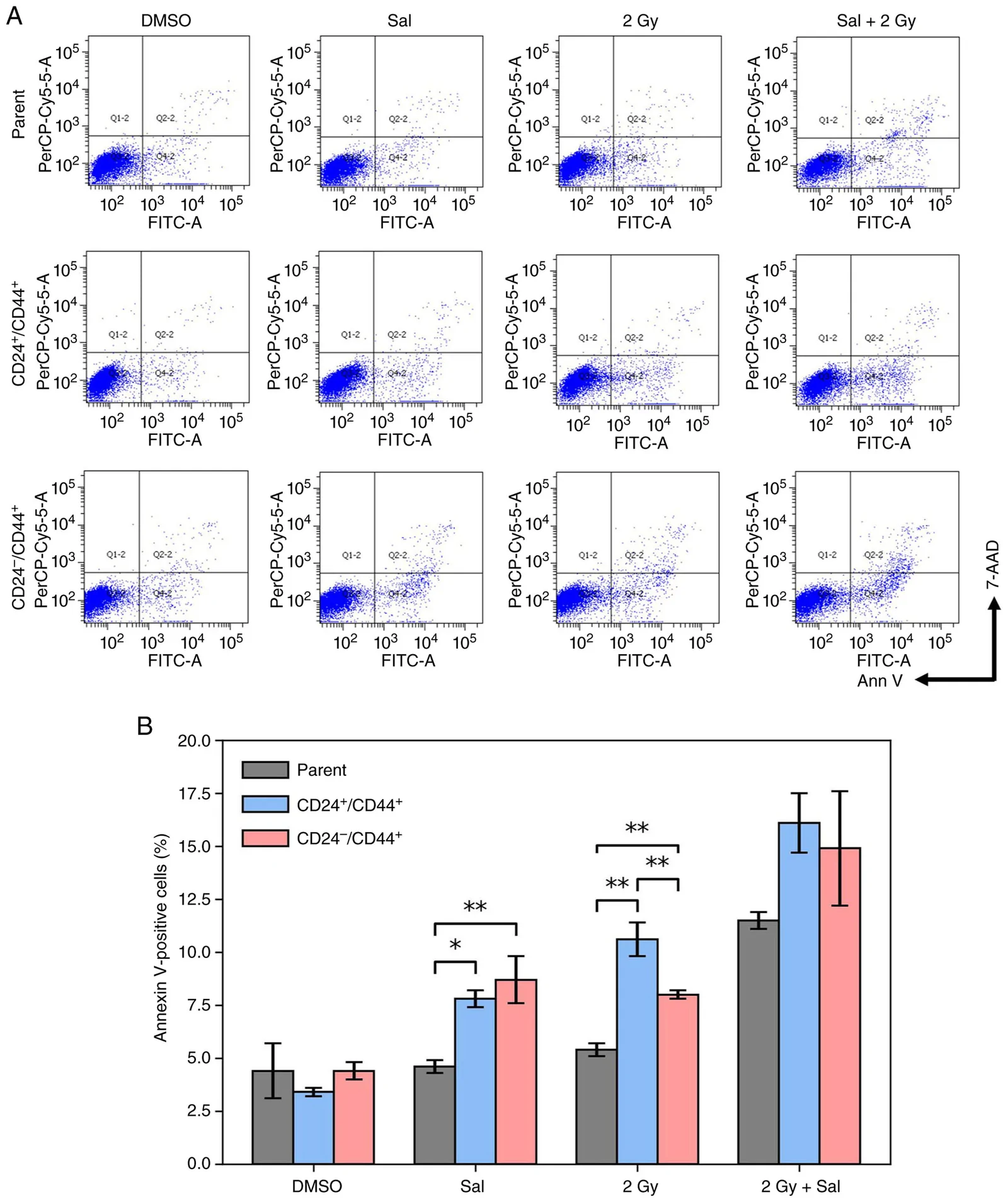
Apoptotic cells treated with Sal and/or 2 Gy irradiation. (A) Representative cytograms of apoptotic cells 24 h following treatment with Sal and/or 2 Gy irradiation. (B) Annexin V-positive fractions were defined as the apoptotic fractions. Values are presented as the mean ± standard deviation. ^*^P<0.05 and ^**^P<0.01, significant differences. Sal, salinomycin.

The results of apoptosis at 24 h following experimental treatment in the fraction of the CD24^+^/CD44_+_ and CD24^-^/CD44^+^ cells sorted from parental SAS cells are presented in Fig. 5. The addition of Sal significantly increased apoptosis in both fractions of the cells compared with the parental SAS cells (Fig. 5B; P<0.05 vs. CD24^+^/CD44^+^ and P<0.01 vs. CD24^-^/CD44^+^). Following treatment with 2 Gy irradiation alone, the apoptosis of the CD24^+^/CD44^+^ cells was significantly increased compared with the other two groups (P<0.01). These results were consistent with those of the colony formation assay; however, there was no significant difference in the 2 Gy + Sal cell group, and the apoptosis of the CD24^-^/CD44^+^ fraction was significantly increased compared with the parental SAS cells treated with 2 Gy irradiation alone (P<0.01). It should be noted that the apoptosis assay and the clonogenic assay evaluate different biological endpoints. Whereas the clonogenic assay measures the long-term reproductive capacity of clonogenic cells, apoptosis was assessed only 24 h following treatment and may not fully reflect reproductive cell death. Therefore, early apoptosis is not necessarily associated with clonogenic survival.

## Discussion

Several studies have been conducted to specifically target the DNA damage response to achieve selective cancer cell radiosensitization (14). Nevertheless, despite efforts to overcome radioresistance, the underlying mechanisms behind its development are not yet fully understood. CSCs with intrinsic or acquired radioresistance have higher DNA damage repair capabilities than non-CSCs. Considering these biological characteristics, the possibility of CSC recovery between dose fractionations cannot be ignored (15). Therefore, the mechanisms underlying radioresistance in stem cell-like cells must be elucidated.

The results of the present study demonstrated that there was a high number of CD24^-^/CD44^+^ cells among the SAS-R cells. CD24 and CD44 are widely used as markers for CSCs (8). Even in OSCC cell lines, the CD24^-^/CD44^+^ fraction has been reported in CSC-like cells (16). CD24 is a cell surface protein expressed on epithelial cells, and its expression has been confirmed in a variety of cancer types (17). One characteristic of cancer cell populations as they transition to CSCs is epithelial-mesenchymal transition. Therefore, when CSCs increase, the epithelial marker CD24^+^ cells decrease and CD24^-^ cells increase (18). Previous research has demonstrated that the expression of CD44 is associated with prognosis. For example, Kawano *et al* (19) demonstrated that CD44^+^ cells with CSC features were associated with a poor prognosis of patients with head and neck carcinomas. A high expression of CD44 reflects the aggressiveness of tumor cells and the malignancy of head and neck carcinomas (20). Furthermore, CD44^+^ cells are more likely to form lung metastases in mice, likely due to their increased migratory and invasive abilities (21).

Various studies have investigated the mechanisms by which CSCs exhibit resistance to radiation (22). In photon radiotherapy, the majority of radiation-induced DNA damage in cancer cells is caused by free radicals, such as reactive oxygen species (ROS) generated by the ionization of water molecules, that is, the indirect action of radiation. There are a multitude of publications showing that ROS scavengers are upregulated and highly efficient in CSCs of various tumors (22). Tumor cells reduce levels of ROS and protected CSCs from radiotherapy-induced cell death (23). In addition to these mechanisms, some studies have demonstrated that the activation of signaling pathways related to survival, such as Bcl-2 and PI3K/Akt/mTOR, also contributes to CSC radioresistance (24). The results of a previous study by the authors indicated that the double-strand break repair mechanism in radioresistant cells strongly depends on the NHEJ repair function (25).

The mechanism of action of Sal in cancer cells has been studied from various perspectives (26). In a previous study on breast cancer cells (MDA-MB-231), Sal induced apoptosis and autophagy (27). The Sal-mediated ROS production led to mitochondrial dysfunction in MDA-MB-231 cells. Notably, treatment with N-acetyl-L-cysteine, a ROS scavenger, attenuated Sal-induced apoptosis and autophagy (27). In another study, Sal induced mitochondrial dysfunction in cisplatin-resistant breast cancer cells. The analysis of nuclear translocation of pro-survival transcription factors by western blotting revealed a distinct role of p65 (NF-κB) in cisplatin-mediated resistance in breast cancer (28). In chemoresistant populations, Sal significantly induced programmed cell death by inducing G2/M cell cycle arrest and inhibiting the MAPK/PI3K pathways (29). That study demonstrated that Sal induced toxic DNA lesions and prevented subsequent recovery by targeting homologous recombination repair. In the present study, the results of the colony formation assay revealed that the number of SAS-R cells decreased when Sal was added at concentrations of 0.3 µM or higher without radiation. This suggested that Sal was effective against SAS-R cells as they included a high proportion of CSCs.

To date, to the best of our knowledge, there have been no studies on the effects of Sal on radioresistant oral cancer cells. However, Lim *et al* (30) investigated the potential of Sal to enhance radiotherapy and uncovered novel dual functions of this ionophore inducing DNA damage and preventing repair using glioblastoma cells. On the other hand, Zhang *et al* (31) demonstrated that Sal induced apoptosis and G2/M arrest, increased Bax and cleaved caspase-3, decreased Bcl-2 expression, and increased the formation of γ-H2AX nuclear foci in nasopharyngeal carcinoma cells. In addition, a potential mechanism underlying the radioresistance of SAS-R cells has been reported (32). SAS-R cells are more resistant to ferroptosis, an iron-dependent form of cell death, than the parental SAS cells due to lower intracellular Fe^2+^ levels resulting from the higher expression of miR-7-5p. Moreover, Sal has been reported to eliminate CSCs through iron-mediated production of ROS (33) and to overcome the radioresistant of nasopharyngeal carcinoma cells by promoting ROS generation via the inhibition of Nrf2(34). These findings may explain the colony formation assay results obtained herein, in which Sal treatment significantly reduced the survival of SAS-R cells compared with that of SAS cells.

The present study has several limitations. First, all experiments were conducted exclusively *in vitro*, without *in vivo* validation using animal models. Second, the molecular pathways discussed above were not experimentally validated. Finaly, although the cell-sorting experiment provides supportive evidence for the involvement of the CD24^-^/CD44^+^ population in the enhanced response to Sal, it does not directly demonstrate the molecular mechanism responsible for Sal-mediated radiosensitization. Further studies are warranted to elucidate the detailed molecular mechanisms underlying the radiosensitizing effects of Sal and to evaluate its potential as a novel CSC-targeting radiosensitizer.

In conclusion, the present study demonstrates that Sal is effective in reducing the survival of radioresistant OSCC cells, which contain a high proportion of CSC-like fraction. As modern radiotherapy mainly relies on fractionated irradiation, radioresistant cells are considered to contribute to poor clinical outcomes. The findings presented herein suggest that Sal may serve as a potential radiosensitizer by reducing the survival of radioresistant OSCC cells and the CD24^-^/CD44^+^ cell fraction in combination with irradiation.

## Data Availability

The data generated in the present study may be requested from the corresponding author.
